# Glial Cell-Axonal Growth Cone Interactions in Neurodevelopment and Regeneration

**DOI:** 10.3389/fnins.2020.00203

**Published:** 2020-03-10

**Authors:** Michael J. Rigby, Timothy M. Gomez, Luigi Puglielli

**Affiliations:** ^1^Department of Medicine, University of Wisconsin-Madison, Madison, WI, United States; ^2^Neuroscience Training Program, University of Wisconsin-Madison, Madison, WI, United States; ^3^Waisman Center, University of Wisconsin-Madison, Madison, WI, United States; ^4^Department of Neuroscience, University of Wisconsin-Madison, Madison, WI, United States; ^5^Geriatric Research Education Clinical Center, Veterans Affairs Medical Center, Madison, WI, United States

**Keywords:** glia, growth cone, axon, neurodevelopment, cell-cell interaction, spinal cord injury

## Abstract

The developing nervous system is a complex yet organized system of neurons, glial support cells, and extracellular matrix that arranges into an elegant, highly structured network. The extracellular and intracellular events that guide axons to their target locations have been well characterized in many regions of the developing nervous system. However, despite extensive work, we have a poor understanding of how axonal growth cones interact with surrounding glial cells to regulate network assembly. Glia-to-growth cone communication is either direct through cellular contacts or indirect through modulation of the local microenvironment via the secretion of factors or signaling molecules. Microglia, oligodendrocytes, astrocytes, Schwann cells, neural progenitor cells, and olfactory ensheathing cells have all been demonstrated to directly impact axon growth and guidance. Expanding our understanding of how different glial cell types directly interact with growing axons throughout neurodevelopment will inform basic and clinical neuroscientists. For example, identifying the key cellular players beyond the axonal growth cone itself may provide translational clues to develop therapeutic interventions to modulate neuron growth during development or regeneration following injury. This review will provide an overview of the current knowledge about glial involvement in development of the nervous system, specifically focusing on how glia directly interact with growing and maturing axons to influence neuronal connectivity. This focus will be applied to the clinically-relevant field of regeneration following spinal cord injury, highlighting how a better understanding of the roles of glia in neurodevelopment can inform strategies to improve axon regeneration after injury.

## Introduction

The developing nervous system is a complex *milieu* of neurons, glial support cells, extracellular matrix, and budding vasculature that elegantly organizes into a highly stereotyped structure. Combinatorial actions of many well-characterized extracellular and intracellular events guide axons to their target locations, which is heavily influenced by tissue mechanics, bound and soluble secreted chemical factors, and cell-cell interactions. The interactions between axonal growth cones and surrounding cells within the developing nervous system is an important component of neurodevelopmental biology but is often not well characterized due to the challenges with observing these transient cellular interactions *in vivo*.

Glial cells in the developing nervous system have many supportive physiological functions, like maintaining solute and nutrient homeostasis, as well as migratory and synaptic development and maintenance roles, such as assisting neurons in reaching their target locations (Chotard and Salecker, 2004; Chao et al., 2009). Glial cells regulate axon growth cone pathfinding, such as acting as guidepost cells along migratory routes creating cellular boundaries as “no-go” zones; they can also assist in proper targeting of axon terminals by serving as transient synaptic partners. Glia have the ability to either directly interact with growing axons through cell adhesion or indirectly by secreting factors that modulate the local microenvironment to promote or inhibit axon growth. Many glial subtypes have been demonstrated to directly impact axon growth and guidance including microglia (Reemst et al., 2016), oligodendrocytes (Chen et al., 2002a; Gang et al., 2015), astrocytes (Cavalcante et al., 2002; Liu R. et al., 2015), Schwann cells (Thompson and Buettner, 2006; De Luca et al., 2015), neural progenitor cells (Merianda et al., 2017), and olfactory ensheathing cells (Windus et al., 2010), and they can either promote or inhibit growth depending on the circumstance. Expanding the knowledge of how different supporting cell types may directly or indirectly interact with growing axons will offer a deeper understanding of the intercellular crosstalk occurring in neurodevelopment, as well as provide clinically useful information, such as identifying potential drugs to modulate neuron regeneration (De Luca et al., 2015; Gang et al., 2015).

This review will provide a brief overview of the current knowledge about glial cell contributions to axon growth and guidance, specifically focusing on how glial cells directly interact with growing axons to influence neuronal connectivity. A selection of examples where glial cell-axonal growth cone interactions are shown to play a crucial role during neurodevelopment will be critically discussed. This basic science background is then linked with translational work targeting glia to promote regeneration following spinal cord injury, highlighting how a better understanding of the role of glia in neurodevelopment can inform strategies to improve axon regeneration after injury.

## Neuron and Glia Development: Right Place at the Right Time

Glial cell development in the central nervous system (CNS) and peripheral nervous system (PNS) often occurs alongside neuron development and maturation as many glial subtypes originate from a common precursor stem cell (Cameron and Rakic, 1991; Lee et al., 2000; Pinto and Gotz, 2007; Grabel, 2012). Therefore, many glial cell subtypes are present at the right time and place to directly impact axon growth and guidance.

Microglia are the only glial cell to enter the CNS from the periphery, doing so well before CNS-resident glia differentiate (Reemst et al., 2016). Unlike other glial cell types, microglia possess a unique origin, the yolk sac (Samokhvalov et al., 2007; Prinz and Priller, 2014), which in mice can be detected as early as embryonic day 7.5 (E7.5) (Kierdorf et al., 2013) and invade the CNS beginning around E8-9 using specific matrix metalloproteinases (Alliot et al., 1999; Kierdorf et al., 2013). Colonization of the brain has been observed to occur in two waves with the first being at E8-9 and second at E14-16; both of these events are independent of the vascular system as the cells enter via the meninges or from the ventricles, thus invading the brain parenchyma from both superficial and deep layers (Reemst et al., 2016). In contrast to the adult brain, microglia in the embryonic brain tend to cluster near developing axons (Reemst et al., 2016), such as around the axonal tracts of the subpallium at E14.5 (Squarzoni et al., 2014) and corpus callosum at E15.5-17.5 (Pont-Lezica et al., 2014). This pattern continues in postnatal development with microglia associating with subcerebral, callosal, and hippocampal perforant path-projecting axons (Dalmau et al., 1998; Rochefort et al., 2002; Ishii et al., 2013). The close association between microglia and developing white matter tracts suggests a role in axon growth, guidance, and/or survival during CNS development, which is further strengthened by the accumulating data from studying CNS injury and regeneration.

Astrocytes and oligodendrocytes, the macroglia in the CNS, originate from a common precursor, the radial glial (RG) cell (Cameron and Rakic, 1991; Lee et al., 2000; Grabel, 2012). RG cells appear around E9-10 in mice marking the beginning of neurogenesis, followed by gliogenesis. Derived from neuroepithelial cells, RG cells span the neural tube in the brain and spinal cord with their apical endfeet on the ventricular surface and a single radial process that contacts the basal pial surface. This dynamic cell type undergoes a series of symmetric or asymmetric divisions that either self-renew or begin producing committed postmitotic neurons or glial daughter cells (Huttner and Kosodo, 2005). In the cortex, postmitotic cells migrate toward the pial surface along the radial process to complete differentiation at the appropriate layer (Grabel, 2012), and this glial-guided neural migration is dependent on gap junction adhesions (Elias et al., 2007). Oligodendrocyte precursor cells (OPCs) develop primarily in the ventral neural tube, migrate laterally and dorsally to their proper locations, and continue to differentiate and change morphologically to begin the myelination process (Lee et al., 2000). In the adult brain, oligodendrocytes can also be derived from parenchymal oligodendrocyte progenitor cells as well as adult neural precursor cells from the subventricular zone following a demyelinating disease (Xing et al., 2014). Astrocytes develop later than oligodendrocytes and are primarily born in the dorsal neural tube; they populate developing white and gray matter and serve a myriad of functions including maintenance of solute homeostasis, axon guidance, and synaptic formation (Mason et al., 1988; Giaume and Venance, 1998; Bacci et al., 1999; Cavalcante et al., 2002; Liu R. et al., 2015). Additionally, astrocytes are classified into two subtypes: (1) fibrous astrocytes within white matter and (2) protoplasmic astrocytes within gray matter (Kimelberg, 2010); recent studies have demonstrated differences in their propensity to promote neurite growth (Liu R. et al., 2015), which is discussed in greater detail below. It is important to note that there are well-documented differences in radial glial development in the cortex versus spinal cord. For example, radial glia are vital in regulating vascular patterning within the spinal cord (Matsuoka et al., 2016), and astrocytes derived from regionally-distinct sites exhibit unique molecular signatures (Bachoo et al., 2004; Yoon et al., 2017; Bradley et al., 2019). What remains to be fully elucidated is how knowledge of these differences can be utilized for region-specific intervention, such as aiding in regeneration in the cortex versus spinal cord.

In the PNS, glial cells are derived from neural crest cells that differentiate while migrating to their final destination (Jessen and Mirsky, 2005). These migrating neural crest cells form Schwann cell precursors (SCPs) and then immature Schwann cells that begin to associate with axons; an additional branching lineage includes the formation of satellite cells that eventually associate with peripheral ganglia (Le Douarin and Ziller, 1993). The eventual fate of immature Schwann cells is determined by the type of axons they associate with, directing them to become non-myelinating or myelinating Schwann cells. Interestingly, even though SCPs are present during times of perfuse axon extension and development, they are not required for the axons to reach their target location (Grim et al., 1992; Sepp et al., 2001). Nonetheless, several studies have shown Schwann cells can impact axon outgrowth and guidance, which is especially relevant in PNS injury and repair (Thompson and Buettner, 2006; De Luca et al., 2015).

Finally, olfactory ensheathing cells (OECs) are a unique population of Schwann cells that facilitate the replenishment of olfactory neurons (Farbman and Squinto, 1985; Chuah and Au, 1991; Barnett and Riddell, 2004; Windus et al., 2010). This unusual PNS-CNS connection involves the invasion of peripheral olfactory receptor neurons, which originate from the basal stem cells of the olfactory epithelium, into the cribriform plate and olfactory bulb to form synapses with second-order neurons in the glomerular layer (Barnett and Riddell, 2004). OECs are derived from precursor cells within the olfactory epithelium and closely associate with growing axons (Chuah and Au, 1991). Interestingly, the olfactory receptor neurons are continually turning over so new olfactory receptor neurons must be replenished throughout life. The OEC Schwann cells provide permissive substrata for the migration of new olfactory receptor neurons into the olfactory epithelium where new synapses form throughout life (discussed further below).

## Glial Cell-Axonal Growth Cone Interactions: A Selection of Examples

**FIGURE 1:**
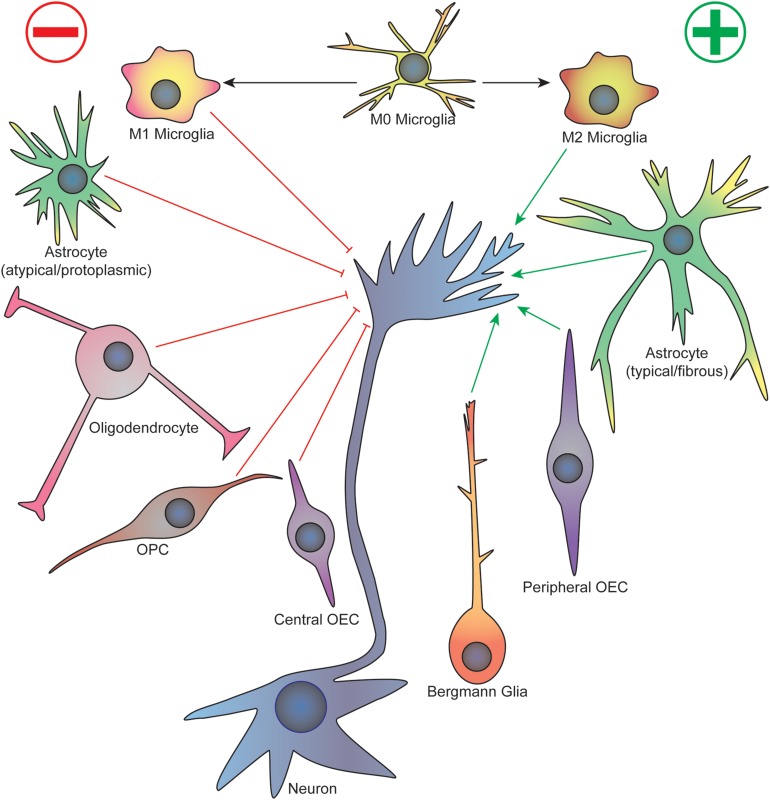
Summary of glial cell-axonal growth cone interactions during neurodevelopment and regeneration. Green arrows represent attractive guidance cues while red represent repellent. See text for description. OPC, oligodendrocyte precursor cell; OEC, olfactory ensheathing cell.

Some of the earliest work studying the cellular events that underlie neurodevelopment established the importance of glia in the growth and guidance of migrating neurons and axons. For example, the first axons to cross the corpus callosum in the developing mouse brain cross a cellular “sling” made up of primitive glial cells suspended below the longitudinal cerebral fissure, which disappears after birth (Silver et al., 1982). These commissural axons also avoid regions containing glial cells, such as the “glial wedge” that express inhibitory axon guidance cues (Shu and Richards, 2001). On the other hand, migrating granule cells in the developing mouse cerebellum follow along vertically oriented Bergmann fibers arising from Golgi epithelial cells, a protoplasmic astrocyte (Rakic, 1971). During *Drosophila* early embryogenesis, three classes of glial cells form an organized pattern at each body segment before axon outgrowth occurs, and these cells enwrap the axon tracts as they migrate (Jacobs and Goodman, 1989). Importantly, loss of peripheral glia in *Drosophila* results in sensory axon stalling and pathfinding defects as they migrate toward the CNS, as well as early migration defects in pioneer motor axons as they cross the CNS/PNS transition zone (Sepp et al., 2001). Although these initial studies relied heavily on fixed sample imaging that provided authors only a static view of specific time points, they provided much of the foundational observations to influence future studies examining the dynamic interface between glia and growing axons. A focused view on specific glial subtypes will be discussed citing important events in specific regions of the CNS and PNS during development (see Figure 1 for a summary).

### Astrocyte-Axonal Growth Cone Interactions

Astrocytes can form a variety of cellular processes that directly interact with growing axons. *In vitro*, both astrocytes and granule neurons form plasma membrane “microspikes” that continually protrude and retract; it is only after contact is made between the astrocyte and granule neuron that the granule neuron microspikes are stabilized, promoting the formation of a neurite that grows over the cell body of the astrocyte (Mason et al., 1988). Using electron microscopy (EM), small, adherent junctions were observed at contact points between the astrocytes and granule neurons. Functional studies demonstrated that these interactions depend on neural cell adhesion molecule (NCAM), N-cadherin, and integrin β1, as blocking the astrocyte-neuron interactions via antisera against these proteins resulted in reduction or elimination of neurite outgrowth onto cultured astrocytes (Keilhauer et al., 1985; Neugebauer et al., 1988; Tomaselli et al., 1988). Interestingly, integrin β1 antisera only blocked E8 but not E14 chick ciliary ganglion neuron outgrowth on rat cortical astrocytes (Tomaselli et al., 1988), unlike chick retinal neurons, which were not affected by age (Neugebauer et al., 1988). Additionally, blocking NCAM also had neuron-type dependent effects: NCAM antisera impacted cerebellar neuron-astrocyte interactions (Keilhauer et al., 1985) and E11 chick retinal neurons interactions with rat cortical astrocytes (Neugebauer et al., 1988), but not chick ciliary ganglion neuron interactions with rat cortical astrocytes (Tomaselli et al., 1988) nor E8 chick retinal neuron interactions with rat cortical astrocytes (Neugebauer et al., 1988). Finally, inhibition of N-cadherin consistently blocked astrocyte-neuron interactions regardless of neuronal type and developmental time point (Keilhauer et al., 1985; Neugebauer et al., 1988; Tomaselli et al., 1988). Together these results suggest a heterogeneity in the molecules used for neuron-glia interactions, with either a neuron type-specific or time-dependent switch over critical time periods.

While heterogeneity in neuronal populations is expected, distinct astrocyte populations also appear to have differential effects on axon development. Liu R. et al. (2015) characterized the development of two astrocyte subpopulations termed “typical” and “atypical” that spontaneously develop when rat primary cortical glial cells were grown *in vitro*. The typical astrocytes were the majority (∼70%) of the astrocytes in the culture, which exhibited a variety of shapes and arrangements, and often co-localized with oligodendrocytes. The atypical astrocytes exhibited a spindle shape with a high cell density arranged in a polarized fashion and covered by fewer oligodendrocytes. When dorsal root ganglia (DRG) neurons were plated on top of this heterogeneous astrocyte population, the typical astrocytes promoted neuron adhesions and neurite growth consistent with prior studies (Keilhauer et al., 1985; Mason et al., 1988; Tomaselli et al., 1988). In contrast, the atypical astrocytes inhibited neuron adhesion and neurite outgrowth without impacting neuron survival. It is plausible that these two astrocyte phenotypes represent the natural heterogeneity of these glial cells in the CNS rather than an artifact of the *in vitro* methods. The atypical astrocytes may form inhibitor barriers in the developing CNS (e.g., glial wedge) and may be related to damaged or reactive astrocytes that have a well-characterized inhibitory effect on neurite growth both *in vitro* and *in vivo* (McKeon et al., 1991, 1999; Wanner et al., 2008). Liu et al. attempted to connect these *in vitro* results to an *in vivo* model by transplanting DRG neurons into either cortical gray matter or corpus callosum white matter (Liu R. et al., 2015). They observed little neurite growth in the cortical gray matter location but robust neurite growth in the corpus callosum. They drew the conclusion that the fibrous astrocytes, which are found within the white matter, are supportive of neurite growth while protoplasmic astrocytes, the subtype found within gray matter, are not. However, since this *in vivo* experimental system does not exclude the influence of all the other differences that exist between the gray and white matter microenvironments, the effects observed on the neurite growth may be completely independent of the astrocytes within the tissue. Furthermore, the results of enhanced neurite growth in the corpus callosum are counterintuitive considering that white matter can have a high content of myelin, which is known to be repulsive to axon growth (discussed below). Clearly an important control experiment is to determine if these findings are reproducible in a rodent model with selective astrocyte ablation, which has been generated in other laboratories (Delaney et al., 1996; Sofroniew et al., 1999; Cui et al., 2001). Nonetheless, follow-up studies to examine the differences between typical and atypical astrocytes *in vitro* are warranted and should be more robustly compared to the fibrous and protoplasmic astrocytes observed *in vivo* using modern molecular techniques, such as single cell expression analysis. These data may provide candidate targets to reprogram inhibitory astrocytes to promote axon growth, which is a highly desired outcome after injury.

Astrocytes also play an active role in assembling connections of GABAergic stellate interneurons within the developing cerebellum (Ango et al., 2008; Chao et al., 2009). Bergmann glia (BG) cells, which are highly polarized astrocytes within the cerebellum, form an elaborate arborization of apical fibers that extend into the cerebellar cortex early in the postnatal brain. The stellate interneurons that reside within the upper half of the cerebellar molecular layer innervate the dendritic shafts of the Purkinje cells that reside below, and they do so by following the BG radial projections. Ango et al. observed strong expression of Close Homolog of L1 (CHL1), a member of the L1 immunoglobulin cell adhesion molecule (L1CAM) family of proteins, within both the BG apical fibers and stellate interneurons during postnatal development. Furthermore, global or BG-specific CHL1 knockout resulted in aberrant growth of stellate interneuron axons with reduced synapse formation on target Purkinje cells. The authors proposed that the disruption in stellate interneuron synapse formation on Purkinje cells may explain the poor motor performance of Chl1^–/–^ mice in the Rotarod test (Pratte et al., 2003). Further probing into the electrophysiologic circuitry aberrations and functional motor differences in Chl1^–/–^ mice would be an interesting follow-up study to solidify the impact of disrupting BG-mediated stellate interneuron axon migration in the cerebellum. However, another research group did not detect changes in the stellate interneuron population within the cerebellum in Chl1^–/–^ mice, rather they observed a decrease in Purkinje cell number, complicating the story (Jakovcevski et al., 2009). Despite loss of Purkinje neurons, Jakovcevski et al., as well as other groups, did not detect motor deficits in Chl1^–/–^ mice (Montag-Sallaz et al., 2002; Jakovcevski et al., 2007; Morellini et al., 2007), reaching the conclusion that cerebellar function is grossly preserved in these mice. These studies suggest that more refined electrophysiological measurements, as well as fine motor tasks and non-motor assessments, should be performed on Chl1^–/–^ mice. While the necessity of stellate interneuron axon guidance on specific adhesion molecules is unclear at this point, it is clear that interactions between astroglia and axon growth cones are important for proper synapse formation within the cerebellum.

### Oligodendrocyte-Axonal Growth Cone Interactions

Oligodendrocytes and oligodendrocyte precursor cells (OPCs) are notorious for their inhibitory effect on axon growth and guidance (Caroni and Schwab, 1988; Fawcett et al., 1989; Bandtlow et al., 1990; McKerracher et al., 1994; GrandPre et al., 2000; Chen et al., 2002a, b; Kottis et al., 2002; Wang et al., 2002; Moreau-Fauvarque et al., 2003; Vourc’h et al., 2003; Goldberg et al., 2004; Pasterkamp and Verhaagen, 2006; Shim et al., 2012; Gang et al., 2015). Early studies examining DRG axon growth in an oligodendrocyte co-culture system revealed that axons avoided growing upon oligodendrocytes unlike other glial cells such as astrocytes. Furthermore, live imaging revealed axonal growth cone stalling and collapse upon membrane contacts with oligodendrocytes (Fawcett et al., 1989; Bandtlow et al., 1990). A number of secreted and membrane proteins produced by oligodendrocytes that are in part responsible for this inhibition include Nogo (GrandPre et al., 2000), myelin-associated glycoprotein (MAG) (McKerracher et al., 1994), oligodendrocyte-myelin glycoprotein (OMgp) (Kottis et al., 2002; Wang et al., 2002), and the semaphorins Sema4D (Moreau-Fauvarque et al., 2003), Sema5A (Goldberg et al., 2004), and Sema6A (Shim et al., 2012). Nogo, MAG, and OMgp comprise the Nogo receptor (NgR) ligand family, which are also referred to as myelin-associated inhibitors (MAIs) that stabilize neuronal structure (Vourc’h and Andres, 2004; Schmandke et al., 2007). Interestingly, expression of many of these inhibitory molecules increases after injury, suggesting that blocking these key targets would improve axon regeneration (Pasterkamp and Verhaagen, 2006). MAIs serve seemingly redundant inhibitory activities toward axon extension, but also exhibit some key differences in function that may be important in neurodevelopment. For example, MAG does not always act as an inhibitor of axon growth as the case when applied to newborn DRG neurons (Mukhopadhyay et al., 1994). Furthermore, myelin preparations from MAG knockout mice do not inhibit neurite elongation or cause growth cone collapse compared to myelin preparations from wild type mice (Bartsch et al., 1995). Additionally, myelin preparations from adult rats actually stimulated axon growth of rodent neural progenitor cells and human induced pluripotent stem cell-derived neural stem cells, which was dependent on interactions with neuronal growth regulator 1 (Negr1) (Poplawski et al., 2018). Therefore, highlighting these differences in MAIs and downstream mediators are important when designing translational neural regeneration applications.

While it is clear that oligodendrocytes function within the adult and injured CNS, there is limited evidence for roles of oligodendrocyte-expressed inhibitors during neurodevelopment while nascent axonal growth cones are searching for their targets. *In vitro* studies on Nogo ligands suggest these inhibitory molecules expressed on oligodendrocytes and myelin may serve to prevent axon sprouting in the adult CNS (Vourc’h and Andres, 2004). Moreover, the abundance of myelin in white matter may be one explanation for its low structural plasticity in contrast to gray matter, as regions of high plasticity tend to have low myelin content (Silver et al., 2015). It is important to note that the developmental time point when myelin becomes abundant is much later than the appearance of nascent axon growth cones; thus, it is unlikely that MAIs play a major role in shaping neuronal networks (McKerracher and Rosen, 2015). Nonetheless, there are numerous studies examining the impact of several inhibitory molecules, especially the semaphorins, on various neurodevelopmental events (Iketani et al., 2016; Wang L. et al., 2017), but these are outside the scope of this review.

Of interest in this review is the neural/glial antigen 2 (NG2) integral membrane proteoglycan expressed by OPCs during neurodevelopment (Chen et al., 2002a). NG2 belongs to the family of chondroitin sulfate proteoglycans (CSPGs) that have a well-known inhibitory effect on axon growth, especially within glial scars. For example, acute treatment of DRG neurons with soluble NG2 induces growth cone collapse. Furthermore, rat ventral spinal cord explants cultured upon 3-dimensional collagen gels with membrane vesicles embedded from NG2-expressing HEK293 cells exhibited reduced neurite length and axon bundling when compared to control conditions (Chen et al., 2002b). Importantly, the authors found high expression of NG2 in the developing rat embryo in areas such as the notochord, perinotochord mesenchyme, lateral mesoderm, base of limb buds, and optic chiasm where segmental patterning was observed. β_III_-tubulin-positive axon bundles were found in regions of low NG2 labeling, suggesting that these axons originally migrated through regions of low NG2 expression. Regions such as the perinotochord mesenchyme have been previously characterized as barriers to axon growth, and the authors suggest that NG2 expression in these regions may limit axon growth, forming repellent boundaries to prevent axon straying. Observing axon extension dynamics in live preparations with *in vivo* two-photon excitation microscopy would be very informative to detect cellular interactions during the development of this circuit. As NG2 can be expressed by a variety of immature cell types (Levine and Nishiyama, 1996) and pericytes (Laredo et al., 2019), this raises the possibility that many of the cells observed *in vivo* in this study are not OPCs nor oligodendrocytes. In fact, the role of NG2-positive glia continues to be heavily debated, and their influence on neurodevelopment and regeneration remains an open question (Silver et al., 2015).

### Microglia-Axon Growth Cone Interactions

Microglia are CNS-resident macrophages that serve a number of important roles in regulating tissue homeostasis, namely phagocytic scavenging, localized immune function, modulation of synaptic transmission, synaptogenesis, and neurotrophic support (Reemst et al., 2016; Henstridge et al., 2019; Rotterman et al., 2019; Wilton et al., 2019). Microglia are implicated in both health and disease, gaining recent attention as a cell type that can be targeted for therapeutic purposes (Tay et al., 2017). Microglia are highly active in autoimmune and injury-related diseases, such as multiple sclerosis and spinal cord injury; thus, understanding their normal physiologic role in development and tissue homeostasis may provide clues for their role in disease states and offer potential targets for intervention.

Some of the hypothesized roles of microglia in axon growth and guidance were originally associated with their phagocytic properties including pathway clearance for developing axons, elimination of transient axonal projections, or clearance of axon growth and guidance factors. More recently, new evidence suggests that microglia may themselves secrete factors that mediate axon growth and guidance (Reemst et al., 2016). Within the mouse embryonic brain, microglia are positioned at decision points along specific axonal tracts rather than associated with vasculature, regions of cell death, or at progenitor zones, where they are typically located postnatally (Squarzoni et al., 2014). During development, microglia are observed near tyrosine hydroxylase (TH)-positive dopaminergic axons as they enter the subpallium. EM revealed that the microglia in the subpallium contained TH^+^ axon fragments within their cytosol, suggesting a phagocytic role. Interestingly, microglial depletion resulted in enhanced growth of the dopaminergic axons within the subpallium, while maternal excess immune activation of microglia resulted in reduced axon growth. Similarly, microglia also play a role in localization of LIM/homeobox 6 (Lhx6)-positive interneurons, which originate in the subpallium, migrating tangentially into the neocortex and eventually migrating radially into the cortical plate. However, when microglia were depleted, Lhx6-interneurons entered prematurely into the cortical plate and had more diffuse localization in layer V, which persisted postnatally (Squarzoni et al., 2014). Furthermore, Cx3cr^–/–^ mice exhibited expansion of TH^+^ axons in the subpallium, as well as diffuse localization of Lhx6^+^ interneurons within the neocortex as a result of impaired microglia-neuron communication. Taken together, these data provide convincing *in vivo* evidence that microglial activity plays an important role in limiting axon outgrowth of dopaminergic neurons within the subpallium, as well as interneuron distribution within the neocortex. Consistent with these findings, microglia also play a role in axon bundling and maturation, as microglial activation or knock-down resulted in defasciculation (Pont-Lezica et al., 2014). Transcriptome analysis revealed a down-regulation of genes involved in “nervous system development and function,” such as *Sema3C*, *PlnxA2*, and *Vcan*, in both activated or defective microglia. These data demonstrate that microglia are important players in the wiring of the mouse forebrain, and both hyperactivation, as well as defective microglia, are likely detrimental to axon growth and guidance.

In the postnatal brain, microglia continue to be tightly associated with certain populations of maturing neurons. For example, in cats, microglia cluster within the white matter beneath cortical areas A17/A18, which contain juvenile exuberant callosal projection neurons that project to the contralateral A17/A18 by crossing through the corpus callosum (Rochefort et al., 2002). With normal rearing (NR), many of these projections are eliminated, which is associated with amoeboid-like microglia consistent with phagocytically active cells. However, in cats raised with monocular deprivation (MD condition) from birth, most projections are stabilized and retained in the adult animal, and microglia exhibit more ramified morphology consistent with a resting, quiescent state (Rochefort et al., 2002). These data demonstrate that microglia function is regulated by postnatal visual experience, which indicates that there are likely close interactions between microglia and axons of the visual neurons. In addition, since microglia exhibited more phagocytic-like appearance in the condition where juvenile exuberant callosal projections are expected to degenerate, the microglia may be an important mediator of this postnatal axonal elimination. In contrast, another study demonstrated that microglia in mice are vital for the support and survival of callosal projection neurons through the secretion of the trophic factor IGF1 (Ueno et al., 2013), which would be in opposition to the conclusions drawn above. These seemingly contradictory results are common throughout studies of microglia in development and most likely represent their diverse function, as well as heterogeneity in methods and animal models employed.

Additional *in vitro* evidence demonstrates that microglial activation can be inhibitory to axon growth and guidance. Microglia activated by lipopolysaccharide (LPS) inhibited neuron outgrowth and induced growth cone collapse (Kitayama et al., 2011). Importantly, this effect was only observed when activated microglia and neurons were co-cultured in the same dish; culturing these two populations of cells in a transwell system, which prevents direct contact but allows for continuous bathing media, resulted in no changes in neurite outgrowth or growth cone collapse. This result suggests that the inhibitory effect of activated microglia on axon growth was not due to a secreted factor but rather direct contact via adhesion molecule or phagocytic interaction. The inhibitory effect was subsequently attributed to activated microglia expressing *repulsive guidance molecule a* (RGMa), a glycosylphosphatidylinositol (GPI)-linked glycoprotein that has been previously demonstrated to induce growth cone collapse of retinal axons (Monnier et al., 2002). Addition of RGMa-blocking antibodies or siRNA-mediated knockdown of RGMa in the activated microglia blocked their inhibitory effects on neurite outgrowth and growth cone collapse. Similar effects were observed when the activated microglia were treated with minocycline, a tetracycline antibiotic that was shown to decrease expression of RGMa. Taken together, these results indicate that activated microglia express RGMa and directly inhibit axon growth in a contact-dependent fashion, which is a potential molecular target to use in regeneration therapies.

### Olfactory Ensheathing Cell-Axonal Growth Cone Interactions

Olfactory neurons are unique as they are continuously turned over so new axons must enter the CNS from the periphery throughout life. Olfactory neuron axons are supported by olfactory ensheathing cells (OECs) in both the periphery and within the olfactory bulb, which both aid in the growth and guidance of olfactory axons as well as surround groups of olfactory axons to enhance electrical conduction (Van Den Pol and Santarelli, 2003). Cell surface molecules on OECs promote axon growth, which may prove useful for regeneration after CNS injury. In the developing olfactory nerve, OECs pioneer the path for olfactory neuron axons, extending their cellular processes as much as 15 microns ahead of the axon growth cone, and olfactory processes never extend ahead of the OECs (Tennent and Chuah, 1996). *In vitro*, olfactory neurites prefer to grow upon OECs rather than surface polylysine, often leaving the surface completely to grow on top of the OECs (Van Den Pol and Santarelli, 2003). Interestingly, live imaging reveals that shortly after an axon growth cone contacts an OEC, the growth cone appears to become a passive partner remaining adherent to the migrating OEC. For example, if the OEC moves toward the neuron cell body, the neurite process will shorten as a result. Moreover, the attached axon growth cone follows their partner OEC even when the cell retracts to divide, after which the growth cone remains adherent to one daughter cell after cytokinesis. The adhesion molecules mediating this interaction include NCAM, polysialic acid (PSA)-NCAM, and N-cadherin that are expressed on the surface of OECs during all developmental stages (Miragall et al., 1989; Key and Akeson, 1990; Franceschini and Barnett, 1996; Fairless et al., 2005; Su and He, 2010). Studies performed *in situ* show that cerebellar granule cells seeded on the surface of olfactory mucosa and bulb slices preferentially grow within regions dense in OECs, such as the ventral nerve layer and lamina propria (Van Den Pol and Santarelli, 2003). These findings represent the preferable interaction between axons and OECs that supports neurite growth and introduces the unique concept that olfactory neuron axons may ride along OEC cell bodies as they migrate into the CNS. Furthermore, unique OEC populations are hypothesized due to the differences in anatomical and temporal development of the olfactory bulb (Windus et al., 2010). When primary olfactory neurons are co-cultured with central- and peripheral-derived OECs, axons grow in a dispersed pattern with central OECs but fasciculate upon peripheral OECs. Time lapse imaging showed that peripheral OECs preferably adhere to one another while central OECs display much more variable behavior of adhesion, repulsion, or crossover. The physiologic implication of these observed differences is interesting as it relates to the *in vivo* organization of the olfactory system. In the periphery, as the olfactory axons leave the olfactory epithelium, they form fascicles as they merge into the olfactory nerve mediated by OECs, which is mirrored by their behavior *in vitro*. Once in the CNS, the axons defasciculate and sort themselves dependent on their odorant receptor expression. Interestingly, the effects of central OECs *in vitro* suggest that these OECs may promote or even guide this defasciculation process. Semaphorin3A (Sema3A), a membrane-bound, cleavable chemorepellent found on the OEC cell surface, was found to mediate this defasciculating and sorting process as olfactory neuron axons avoided regions with high Sema3A expression. Furthermore, Sema3A-deficient mice exhibited defects in olfactory neuron axon sorting within the olfactory nerve layer, which persisted into postnatal life (Schwarting et al., 2000). Overall, the relationship between OECs and olfactory neurons is an elegant demonstration of how direct glial-neuron interactions can result in changes in axonal growth behavior to have an impact on olfactory circuit development and function.

### Schwann Cell-Axonal Growth Cone Interactions

Beyond astrocytes, microglia, and OECs, there is little evidence that Schwann cells or Schwann cell precursors (SCPs) play a direct role in axon growth and guidance during PNS development. Rather, the functions of SCPs during neurodevelopment are believed to include trophic support of sensory and motor axons and nerve myelination (Jessen and Mirsky, 2005). Early studies indicate that Schwann cells or SCPs do not guide axons to their target, but rather follow behind (Speidel, 1964; Carpenter and Hollyday, 1992; Bhattacharyya et al., 1994; Gilmour et al., 2002), and growing motor neurons are primarily guided by substrata composition with a preference to follow pioneer axons (Tosney and Landmesser, 1985). Specific ablation of Schwann cells or SCPs in the mouse does not affect the number of peripheral motor and sensory axons that are generated nor their ability to reach their targets to form initial synapses. However, axons without Schwann cell support do subsequently withdraw and degenerate before postnatal life (Riethmacher et al., 1997; Wolpowitz et al., 2000; Britsch et al., 2001; Jessen and Mirsky, 2005). Furthermore, specific ablation of boundary cap (BC) cells, which are neural crest-derived SCPs that reside early in neurodevelopment at the CNS:PNS junction, does not affect motor neuron axon exiting the spinal cord, but does cause displacement of their somata into the periphery (Vermeren et al., 2003). These data emphasize the importance SCPs and Schwann cells in trophic support of developing peripheral nerves and their apparent limited direct involvement in initial axon outgrowth and guidance to their peripheral targets. Typically, these studies lack detailed dynamic information about early peripheral axon growth and rely heavily on static images to draw conclusions. It remains to be determined whether the absence of SCPs or Schwann cells results in any initial aberrant axon growth cone migration that is corrected through redundant mechanisms to allow axons to reach their proper targets. Live cell *in vivo* imaging is necessary to elucidate these details in neurodevelopment.

## Molecular Mechanisms of Glial Cell-Axonal Growth Cone Interactions

**FIGURE 2:**
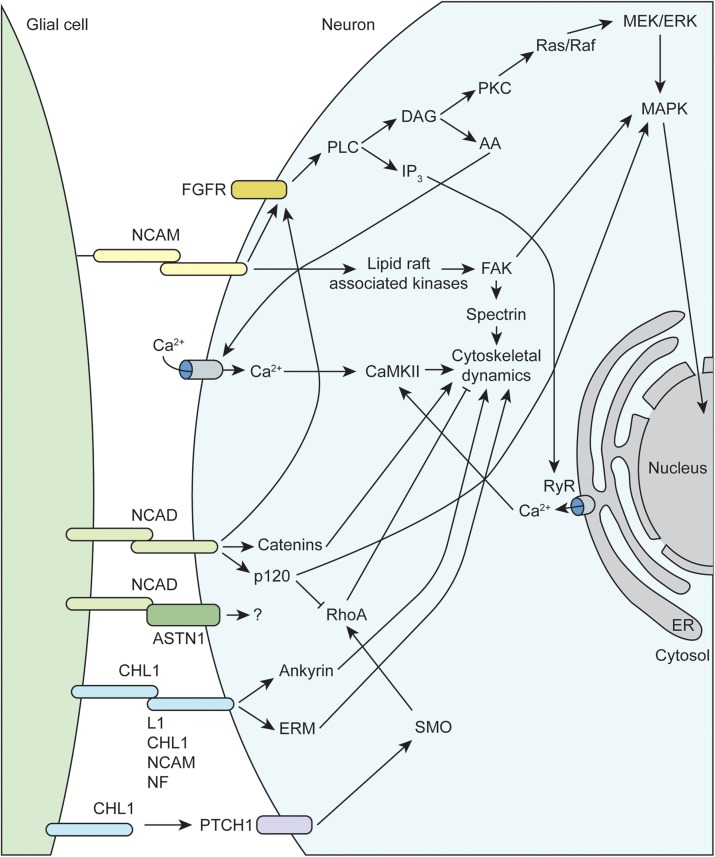
Molecular mechanisms underlying glial-axon growth cone guidance signals. NCAM, neural cell adhesion molecule; FGFR, fibroblast growth factor receptor; CHL1, close homolog of L1; MAPK, mitogen-activated protein kinase; RhoA, Ras family member homolog A; FAK, focal adhesion kinase; NCAD, N-cadherin; NF, neurofascin; ERM, ezrin-radixin-moesin; RyR, ryanodine receptor; PLC, phospholipase C; PKC, protein kinase C; DAG, diacylglycerol; IP_3_, inositol trisphosphate; AA, arachidonic acid; ASTN1, astrotactin-1; PTCH1, protein patched homolog 1; SMO, smoothened; CaMKII, calcium/calmodulin-dependent protein kinase II; ER, endoplasmic reticulum.

**FIGURE 3:**
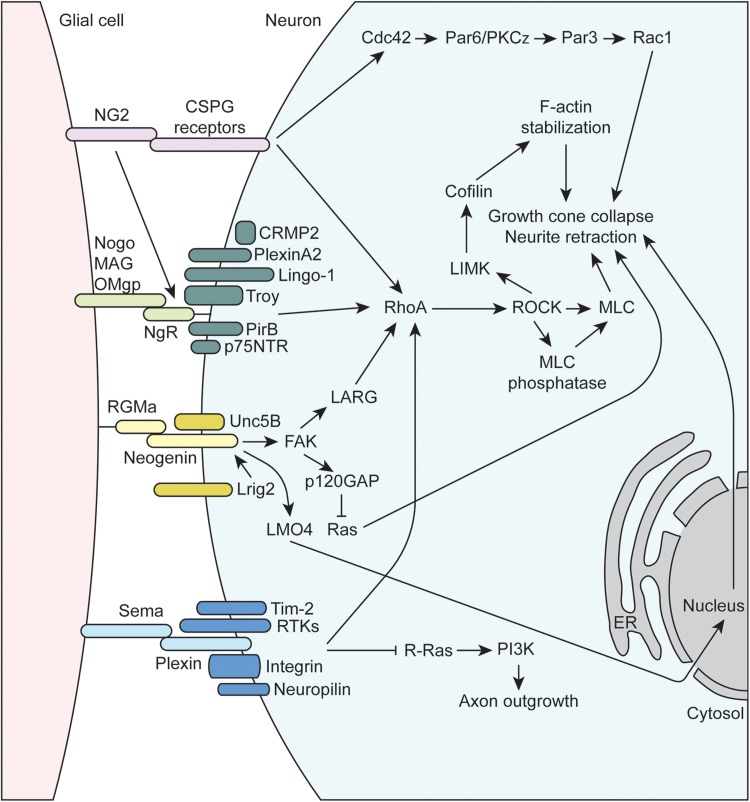
Molecular mechanisms underlying glial-axon growth cone repulsive signals. MAG, myelin-associated glycoprotein; OMgp, oligodendrocyte-myelin glycoprotein; p75NTR, neutrophin receptor; CRMP2, collapsin response mediator protein family-2; RhoA, Ras family member homolog A; RTK, receptor tyrosine kinase; NG2, neuron-glia antigen-2; CSPG, chondroitin sulfate proteoglycan; RGMa, repulsive guidance molecule A; FAK, focal adhesion kinase; LMO4, LIM domain transcription factor; NgR1, Nogo receptor 1; PirB, paired immunoglobulin-like receptor B; Tim-2, T cell immunoglobulin and mucin domain containing 2; Lrig2, leucine rich repeats and immunoglobulin like domains 2; GAP, GTPase activating protein; PI3K, phosphoinositide 3-kinase; LARG, leukemia-associated Rho guanine nucleotide exchange factor; PKC, protein kinase C; LIMK, LIM domain kinase 1; ROCK, Rho-associated protein kinase; MLC, myosin light chain; ER, endoplasmic reticulum.

Multiple families of cell adhesion molecules (CAMs) mediate interactions between glial cells and neurons, with some being specific for certain glial subtypes (see Table 1). Many of these CAMs activate intracellular signaling cascades that converge on common pathways to impact cytoskeletal function and neurite outgrowth (see Figures 2, 3). Many of these CAMs are extensively reviewed (Herron et al., 2009; Siebold et al., 2017; Sytnyk et al., 2017; Chooi and Chew, 2019), thus this section will highlight some of the key players and mechanisms that underlie glial cell-neuron interactions.

**TABLE 1:** Summary of key proteins and mechanisms underlying glial cell-axon growth cone interactions.

Glial protein	Cell type(s)	Neuron receptor(s)	Co-receptors	Signaling mechanism(s)
NCAM120	Astrocyte OEC	NCAM180 NCAM140	FGFR	Lipid raft-associated kinases FGFR signaling

CHL1	Astrocyte	L1 CHL1 Neurofascin NCAM	Sema3A	Ankyrin and ERM recruitment

N-cadherin	Astrocyte OEC	N-cadherin (homophilic)	FGFR ASTN1	Catenins FGFR signaling p120-mediated RhoA inhibition and MAPK activation

Nogo MAG OMgp	Oligodendrocyte	NgR1	p75NTR Troy Lingo-1 PirB PlexinA2 CRMP2	RhoA activation

Sema3A	OEC	Plexins	Neuropilins Integrins	R-Ras inhibition Rho-A activation

Sema4D Sema5A Sema6A	Oligodendrocyte	Plexins	Neuropilins RTKs Tim-2	R-Ras inhibition Rho-A activation

NG2	Oligodendrocyte	CSPG receptors		RhoA activation Par complex alterations

RGMa	Microglia	Neogenin	Unc5B	RhoA activation FAK-mediated Ras inactivation LMO4-mediated transcription

**NCAM, neural cell adhesion molecule; OEC, olfactory ensheathing cell; FGFR, fibroblast growth factor receptor; CHL1, close homolog of L1; MAPK, mitogen-activated protein kinase; MAG, myelin-associated glycoprotein; OMgp, oligodendrocyte-myelin glycoprotein; p75NTR, neutrophin receptor; CRMP2, collapsin response mediator protein family-2; RhoA, Ras family member homolog A; RTK, receptor tyrosine kinase; NG2, neuron-glia antigen-2; CSPG, chondroitin sulfate proteoglycan; RGMa, repulsive guidance molecule A; FAK, focal adhesion kinase; LMO4, LIM domain transcription factor; ASTN1, astrotactin-1; ERM, ezrin-radixin-moesin; NgR1, Nogo receptor 1; PirB, paired immunoglobulin-like receptor B; Tim-2, T cell immunoglobulin and mucin domain containing 2.**

The immunoglobulin superfamily (IgSF) of CAMs, including L1-CAM family members NCAM and CHL1, as well as N-cadherin, mediate much of the attractive interactions between glial cells and axonal growth cones (Figure 2). The L1-CAM family consists of type I transmembrane proteins that have a large extracellular domain, with several immunoglobulin and fibronectin binding domains, as well as a short cytoplasmic tail of approximately 120 amino acids. These CAMs can participate in both homophilic and heterophilic binding interactions either in *cis* or *trans* that allow for a variety of functions depending on differential expression and binding interactions of the cytoplasmic tail (reviewed in Hansen et al., 2008; Herron et al., 2009; Samatov et al., 2016). There are three major isoforms of NCAM arising from alternative splicing that contain identical extracellular domains but variable cytoplasmic tails. The larger 180 kDa isoform (NCAM180) is primarily found in neurons, while the smaller 120 kDa isoform (NCAM120), which is attached to the membrane via a GPI anchor, is primarily found in glial cells (reviewed in Sytnyk et al., 2017). All L1-CAM family members contain the ankyrin-binding motif SFIGQY on their cytoplasmic tails, and the phosphorylation status of the tyrosine residue in this motif mediates binding with ankyrin. Changes in L1-CAM/ankyrin binding regulates coupling of ankyrin-associated proteins to the spectrin cytoskeleton, which can influence growth cone motility (Sytnyk et al., 2017). There is convincing evidence that both L1 and CHL1 can also bind the ezrin-radixin-moesin (ERM) family of proteins, which allow for additional linkage to the actin cytoskeleton. Interestingly, ERM binding to CHL1 was shown to be essential for Sema3A-induced growth cone collapse, providing a mechanism of crosstalk between different CAMs and axon guidance molecules (Schlatter et al., 2008). Similarly, CHL1 has recently been shown to play a role in non-canonical hedgehog signaling via interaction with PTCH1, providing a link to RhoA/ROCK signaling, which is discussed below (Katic et al., 2017). Extracellular NCAM interactions have been shown to activate membrane-linked tyrosine kinases such as Fyn, which can phosphorylate downstream targets like focal adhesion kinase (FAK) and spectrin that impact cytoskeletal dynamics (reviewed in Chooi and Chew, 2019). Finally, multiple studies have demonstrated that NCAM is able to activate fibroblast growth factor receptors (FGFR), which can influence gene expression (via MAPK) and intracellular calcium signaling (Doherty and Walsh, 1996; Niethammer et al., 2002).

Neural cadherin (N-cadherin or NCAD), which belongs to the larger family of calcium-dependent adhesion molecules, promotes cell adhesion through hemophilic binding in *trans*. The intracellular cytoplasmic tail of cadherins associate with p120 catenin complexes to link them to the actin cytoskeleton (reviewed in Hansen et al., 2008; Chooi and Chew, 2019). NCAD and NCAM share some downstream targets, such as activation of FGFR signaling cascades. Additionally, NCAD signaling through p120 both inhibits RhoA activity and activates MAPK, both of which can positively impact growth cone motility (reviewed in Hansen et al., 2008). Recent work has emphasized the importance of co-receptors such as astrotactin (ASTN1) that are important for formation of glial cell-neuron cell adhesions mediated by NCAD (Horn et al., 2018). Finally, endocytic trafficking of NCAD has been shown to regulate neuronal migration and maturation, which depends on Rab GTPase activity (Kawauchi et al., 2010; Shikanai et al., 2011).

The inhibitory or repulsive interactions between glial cells and axon growth cones include the myelin-associated inhibitors (MAIs), semaphorins, NG2, and RGMa (Figure 3). The oligodendrocyte-expressed MAIs include the membrane proteins Nogo, MAG, and OMgp that signal through receptor complexes including the GPI-linked, Nogo-66 receptor NgR1 (reviewed in McKerracher and Rosen, 2015). Importantly, since NgR1 is GPI-linked, co-receptors are required to transmit signaling information within the cell, and the array of co-receptors being expressed can vary for a given neuron. These co-receptors include, but are not limited to, p75NTR, LINGO-1, TROY, PirB, PlexinA2, and CRMP2 (Sekine et al., 2019), and their common intracellular signaling mechanism is through the activation of RhoA, which is a GTPase within the Ras superfamily of proteins. RhoA is in its active form when bound to GTP, which is facilitated by guanine-nucleotide exchange factors (GEFs) that promote the exchange GDP for GTP. GEFs are regulated by a number of complex mechanisms, such as alterations in protein-lipid interactions that can change subcellular localization, release of autoinhibition by a flanking domain or region, and activation by secondary messengers or posttranslational modification (reviewed in Rossman et al., 2005; Bos et al., 2007). RhoA is also regulated via interactions with GTPase activating proteins (GAPs) that promote the conversion of GTP to GDP to inactivate RhoA, as well as guanine-nucleotide dissociation inhibitors (GDIs) that maintain GTPases in an inactive, GDP-bound state. For example, the NgR1 co-receptor p75NTR is cleaved upon ligand binding to NgR1, and the intracellular cleavage product displaces GDI from RhoA leading to its activation (Hasegawa et al., 2004; Domeniconi et al., 2005). Once activated, RhoA binds its downstream effector protein, Rho-associated protein kinase (ROCK) within the α-helical coiled-coil domain, resulting in removal of autoinhibition of ROCK and subsequent phosphorylation of substrate molecules (reviewed in Liu J. et al., 2015). ROCK phosphorylates a number of important targets that impacts axon guidance. For instance, ROCK promotes actin contractility by phosphorylating myosin light chain (activate) and myosin light chain phosphatase (inactivate), which can lead to growth cone collapse and neurite retraction. ROCK also phosphorylates LIM kinase (LIMK), which phosphorylates cofilin, an actin-binding protein responsible for depolymerization of actin filaments. Note that when phosphorylated, cofilin is inactivated, resulting in stabilization of actin filaments. As a result, activation of the RhoA/ROCK signaling pathway stabilizes actin filaments, which provides a substrate for ROCK-activated myosin based contractility, resulting the inhibition of axon growth.

Several other inhibitory axon guidance cues expressed by certain glia signal through the RhoA/ROCK pathway. For example, several of the previously discussed molecules above including RGMa, NG2, and many of the plexins (semaphorin receptors) signal through the RhoA/ROCK pathway to inhibit axon growth and guidance. RGMa is part of the larger family of GPI-linked repulsive guidance molecules (RGMs) that signals through interactions with the type 1 transmembrane protein neogenin (reviewed in De Vries and Cooper, 2008; Siebold et al., 2017). RGMa can act *in trans* to promote the formation of neogenin receptor dimers, which initiates downstream signal transduction. The co-receptor Unc5B, a member of the netrin family, was found to interact with neogenin, which activates downstream signaling to the leukemia-associated Rho GEF (LARG) (Hata et al., 2009). RGMa-induced tyrosine phosphorylation of LARG by FAK has also been found to be necessary for the activation of RhoA and growth cone collapse. Interestingly, the co-expression of Lrig2 adds another layer of regulation to the RGMa-neogenin signaling cascade. Lrig2 association with neogenin prevents premature extracellular cleavage and inactivation of neogenin by A disintegrin and metalloprotease 17 (ADAM17), and RGMa disrupts the Lrig2-neogenin interaction allowing for the cleavage to occur (Van Erp et al., 2015). Thus, Lrig2 co-expression allows neurons to remain RGMa-sensitive by preventing the premature cleavage of neogenin, allowing for subsequent downstream signaling through RhoA. Several other non-RhoA-dependent mechanisms have also been shown to be important in RGMa signaling. For example, FAK has been shown to activate p120GAP, leading to the inactivation of Ras and subsequent growth cone collapse (Endo and Yamashita, 2009). Additionally, RGMa binding neogenin also promotes the cleavage of the neogenin intracellular domain by γ-secretase. Released intracellular cytoplasmic neogenin associates with the transcriptional co-activator LIM-only protein 4 (LMO4) and affects downstream gene expression that mediates growth cone collapse (Schaffar et al., 2008; Banerjee et al., 2016). NG2 also signals to RhoA by interaction with a number of CSPG receptors such as Protein Tyrosine Phosphatase σ (PTPσ), NgR1, and NgR3 (reviewed in Ohtake and Shuxin, 2015). Additional mechanisms of NG2-mediated growth inhibition exist such as signaling through Cdc42 and atypical protein kinase C (PKCζ), which alters Par complex function (Lee et al., 2013).

Semaphorins, a large family that includes GPI-linked and membrane-bound proteins that play critical roles in axon growth and guidance, signal through both RhoA-dependent and independent pathways (reviewed in Liu and Strittmatter, 2001; Negishi et al., 2005; Derijck et al., 2010; Hota and Buck, 2012). Semaphorin signaling is mainly mediated by plexin receptors, a family cell-surface, transmembrane proteins with four subfamilies (PlexinA-D) in mammals. Additional plexin co-receptors like neuropilins, receptor tyrosine kinases (RTKs), and integrins mediate ligand binding and additional downstream signaling within target cells, allowing for diverse semaphorin-mediated signaling. All plexins contain a conserved intracellular GAP homology domain that can directly activate the GTPase activity of multiple GTPase protein families. For example, the plexin-A and B GAP homology domains inactivate R-Ras, resulting in reduced PI3K and integrin signaling (Hota and Buck, 2012). Plexins also contain a Rho binding domain (RBD) that can interact with Rho family GTPases in a number of ways to affect a complex network of downstream proteins (reviewed in Hota and Buck, 2012). Plexins can signal to RhoA by mediating the activity of GEFs, such as LARG, as well as many other possibilities as described in the above reviews.

## Modulating Glial Cell-Axonal Growth Cone Interactions to Aid in Regeneration

One major motivation to improve our understanding of the roles glia play in axon growth and guidance during neurodevelopment is to direct those developmental principles to improve regeneration of the CNS or PNS after injury. For example, glia are known to mediate regeneration following spinal cord injury (SCI) (Cregg et al., 2014; Silver et al., 2015; Jin and Yamashita, 2016). This section aims to provide a few examples showing how the neurodevelopmental discoveries influenced the field of SCI for translational purposes (Table 2).

**TABLE 2:** Pharmaceuticals targeting glial-neuron interactions under study for spinal cord injury.

Glial protein target	Cell type	Intervention	Clinical trial	Phase	Status	References
Nogo	Oligodendrocyte	ATI355/NG-101 (Novartis)	NCT00406016 NCT03935321 EudraCT2016-001227-31	I II II	Complete Ongoing Ongoing	Kucher et al., 2018

Nogo MAG OMgp	Oligodendrocyte	AXER-204	NCT03989440	I/II	Ongoing	

RGMa	Microglia	Elezanumab (ABT-555) Minocycline	NCT02601885 NCT03737812 NCT03737851 Not announced NCT00559494 NCT01828203	I (MS) II (MS) II (MS) I (SCI) II (SCI) III (SCI)	Completed Ongoing Ongoing N/A Completed Ongoing	Casha et al., 2012

*MAG, myelin-associated glycoprotein; OMgp, oligodendrocyte-myelin glycoprotein; RGMa, repulsive guidance molecule A. *The above clinical trial information was acquired from the United States (clinicaltrials.gov) and European Union (clinicaltrialsregister.eu) databases.**

In an injured state, cytokines, cell fragments, and nucleic acids contribute to differentiation of CNS microglia into “classically activated” M1 or “alternatively activated” M2 subtypes (Silver et al., 2015). This decision may be influenced by a number of local factors such as interleukin-4 (IL-4) (Francos-Quijorna et al., 2016) and hemopexin (Han et al., 2018). M1 activated microglia are generally viewed as pro-inflammatory and neurotoxic, promoting axon dieback (see below). M2 activated microglia are anti-inflammatory and neuroprotective, secreting neurotrophic factor and promoting axon regeneration. For example, microglia have been shown to encourage axon elongation and presynaptic site formation following pyramidal tract section (Jiang et al., 2019) as well as promote plasticity following lesions within the visual pathways (Chagas et al., 2019). However, this binary system represents an oversimplification of microglial function. For example, a variety of cytokines are able to promote both M1 and M2 phenotypes, which adds mechanistic uncertainty into the divergent roles of M1 and M2 microglia. Nonetheless, this model provides a framework that is relevant to SCI and categorizes the multiple roles microglia may have on axon growth and regeneration.

Work described above demonstrated that deficiency of Cx3cr1 (Cx3cr^–/–^), the microglial-specific fractalkine chemokine receptor, improved axonal growth of dopaminergic neurons in the subpallium (Squarzoni et al., 2014). Importantly, Cx3cr^–/–^ microglia compared to WT do not exhibit an activated, M1-type morphology when stimulated with the inflammatory mediators interferon-γ (INF-γ) and LPS (Freria et al., 2017). Instead, they remained in an unstimulated, M0 “reparative” phenotype with neurotrophic potential as they expressed higher amounts of TGF-β, IGF-1, and FGF2 compared to WT microglia. After SCI, Cx3cr^–/–^ mice exhibited greater regeneration of serotonergic axons, especially in the ventral horn. This may be partially attributed to the creation of a microenvironment that promoted the differentiation and survival of NG2-positive glia, which include OPCs, as greater numbers closely associate with growing axons in the Cx3cr^–/–^ mice following SCI. The above data, when taken together, suggest that modulating CX3CR could be a therapeutic strategy to enhance axonal regeneration after injury through a mechanism of improved microglial support. Indeed, inhibition of CX3CR via a small molecule or monoclonal antibody has shown promise in other inflammatory conditions, such as atherosclerosis (Poupel et al., 2013), rheumatoid arthritis (Nanki et al., 2004), and multiple sclerosis (Wollberg et al., 2014). Therefore, the next logical step would be to try these interventions in a SCI clinical trial.

RGMa was identified as a major molecule involved in the inhibitory effect microglia exert on growing axons (Kitayama et al., 2011). In a mouse model of SCI, minocycline treatment, which decreases microglial RGMa expression, reduced the accumulation of microglia in the site of injury with a subsequent reduction in axonal dieback in injured corticospinal neurons. Furthermore, intrathecal administration of an antibody against RGMa in a rat SCI model promoted axonal growth and functional recovery, which may be attributed to invasion of microglia and/or macrophages in the site of injury (Hata et al., 2006). Another group developed a systemically administered, human monoclonal antibody against the N-terminus of RGMa, which both neutralized RGMa as well as prevented the RGMa receptor Neogenin from associating with lipid rafts, which is essential for its downstream functions (Mothe et al., 2017). The authors demonstrated that this treatment promoted neuron survival, corticospinal tract axonal regeneration, and improvement in motor function and gait. As such, inhibition of RGMa/Neogenin shows promise in improving clinical outcomes for SCI. In fact, AbbVie Inc. has developed the human anti-RGMa antibody elezanumab (ABT-555) that is currently in Phase II clinical trials for Multiple Sclerosis (NCT03737812 and NCT03737851) with plans to start a Phase I clinical trial for spinal cord injury (see abbvie.com). Additionally, many other pre-clinical studies have demonstrated the beneficial effects of the RGMa-suppressing antibiotic minocycline in SCI (Lee et al., 2003; Wells et al., 2003; Stirling et al., 2004; Festoff et al., 2006; Wang Z. et al., 2017), and a Phase II clinical trial suggested that there may be improvement after acute SCI (Casha et al., 2012). There is an ongoing Phase III clinical trial (NCT01828203) that was expected to complete in June of 2018, but no results have been posted.

In addition to microglia, other glial cells negatively impact CNS injury with the formation of the well-known “glial scar” that is detrimental to axon regeneration. For example, astrocytes react and form a dense barrier around the lesion, stromal cells invade and form fibrous connective tissue with dense collagen and CSPG deposition, and OPCs proliferate and surround dystrophic axons (Pasterkamp and Verhaagen, 2006; Cregg et al., 2014; Dias et al., 2018). Efforts to prevent or dissociate the glial scar have been shown to improve axon regeneration (for example, see Rosenzweig et al., 2019). However, the biology of the glial scar is much more nuanced, and oversimplification of this complex healing and regeneration process can hinder advances in the field (Bradbury and Burnside, 2019). Therefore, a detailed understanding of the players involved, including the glial subtypes, can provide additional clues for intervention. As mentioned above, the expression of many inhibitory molecules such as the MAIs increase following injury, such as Nogo-A (Hunt et al., 2003), which signals through NgR/RhoA pathway to inhibit axon outgrowth. Four Nogo receptors have been identified (NgR1, NgR2, NgR3, and PirB), and Ngr1^–/–^; Ngr2^–/–^; Ngr3^–/–^ mice exhibit improved axon regeneration following optic nerve crush injury (Dickendesher et al., 2012). Furthermore, knockout of Nogo-A in mice improved axon regeneration past the lesion following dorsal spinal cord hemisection, an effect that was not observed in MAG or OMgp knockout mice (Cafferty et al., 2010). Consistently, intrathecal administration of a Nogo-A neutralizing antibody following SCI resulted in enhanced axon growth and collateral sprouting in both rats and non-human primates (Merkler et al., 2001; Freund et al., 2006). These results led to a phase I trial (NCT00406016), which was completed in 2011 to assess the safety of intrathecal administration of ATI355 (Novartis), a recombinant human antibody directed against Nogo-A that was well-tolerated in patients with acute SCI (Kucher et al., 2018). Currently there are two ongoing phase II trials (NCT03935321 and EudraCT2016-001227-31) as a follow-up to this study, with no results posted. An additional approach to antagonize the action of MAIs is via administration of a soluble NgR fragment that can disrupt neural NgR signaling and promote axon regeneration (Fournier et al., 2002). Intrathecal administration in a rat SCI model showed promising results as demonstrated by increased axon sprouting, electrical conduction, and locomotion (Li et al., 2004). To translate this finding into humans, the drug AXER-204 (ReNetX Bio, Inc.), which is a soluble decoy for MAIs like the one previously mentioned, is being used in a current phase I/II trial for patients with chronic SCI (NCT03989440).

In addition to the identification of novel drug targets, pre-clinical studies investigating glial-axon growth cone interactions have led to the notion of implanting growth-promoting glia into the site of injury. For example, OECs can be harvested from the olfactory blub, cultured *in vitro*, and injected into SCI sites. In rodent studies, transplantation of OECs resulted in enhanced axon regeneration and even functional recovery in several animals (Ramon-Cueto et al., 2000; Keyvan-Fouladi et al., 2003; Li et al., 2003). OEC transplants provided several benefits for regenerating axons, such as functioning as a physical substrate for axon growth and secretion of soluble factors that enhanced neurite sprouting (Chung et al., 2004). Interestingly, similar behaviors were observed in spinal cord as seen in the *in vitro* models. For example, some neurons traveled along OECs as they migrated into the injury tract. This may provide a means to avoid the inhibitory signaling molecules that become enriched at injury sites, as regenerating axons may associate with OECs as they migrate through the forming glial scar. Several small clinical trials have been completed in humans (for example, NCT01327768 and NCT01231893) with mixed results, often challenged by small sample sizes and technical difficulties with cell extraction and transplantation (Tabakow et al., 2013, 2014; Wang et al., 2016). There are currently two clinical trials actively recruiting for participants that aim to optimize the OEC harvesting procedure and implant them into patients with SCI (NCT02870426 and NCT03933072). In addition to OECs, spinal cord neural stem cell (NSC) grafts are also being developed with the potential to reconstitute components of the damaged spinal cord. Spinal cord NSCs derived from human pluripotent stem cells and transplanted into lesioned rat spinal cords develop into both neuron and astrocyte lineages, which are able to integrate into the spinal cord circuitry with improvements in functional outcomes (Kumamaru et al., 2018; Lien et al., 2019). Therefore, spinal cord NSCs are an additional cell-based therapy that show promise for translation into humans. With improvements in culturing and transplantation techniques, as well as the potential benefits from combined therapies with different mechanisms of action, there is great promise that the long history of research in glial-axonal growth cone interactions will prove worthwhile to aid in axon regeneration after SCI in human patients.

## Conclusion

In the CNS and PNS, many glial cell types are able to affect axon growth and guidance, which has an impact on neuronal wiring in adulthood as well as the outcomes of several disease states, such as SCI (see Figure 1). Promising therapeutics are being developed because of the advances in knowledge of glial function in neurodevelopment, which shows the importance of further development in this area of pre-clinical research. As more specific molecular tools and labeling techniques become readily available, better correlation can be drawn from the observational data of anatomic distribution and morphology of glia with developing axonal tracts. For example, with improvements in live imaging *in vivo*, much of these developmental events will become accessible to view in real time, which could promise a plethora of important information on glial-axon growth cone interactions (Wu et al., 2013). Furthermore, with advances in cell culture techniques, such as with the multi-compartment neuron-glia co-culture platforms (Park et al., 2012), as well as widespread availability of genome editing and human stem cell differentiation protocols, more streamlined and high-throughput screening of potential drug targets will be available (Gang et al., 2015). Finally, techniques to specifically target glial populations are being further refined with application to both non-human primate models and humans (Juttner et al., 2019). In the end, there will always be a balance between the promoting and inhibiting effects of glia on growing axons, and there are likely benefits to precise molecular reprogramming of glia for both neuroprotective and regenerative applications.

## Author Contributions

MR wrote the manuscript. TG and LP revised the manuscript.

## Conflict of Interest

The authors declare that the research was conducted in the absence of any commercial or financial relationships that could be construed as a potential conflict of interest.
